# (2*E*)-1-(2,4-Dimethyl­quinolin-3-yl)-3-(thio­phen-2-yl)prop-2-en-1-one^1^

**DOI:** 10.1107/S1600536811031485

**Published:** 2011-08-11

**Authors:** R. Prasath, P. Bhavana, Seik Weng Ng, Edward R. T. Tiekink

**Affiliations:** a Department of Chemistry, BITS, Pilani – K. K. Birla Goa Campus, Goa 403 726, India; b Department of Chemistry, University of Malaya, 50603 Kuala Lumpur, Malaysia; c Chemistry Department, Faculty of Science, King Abdulaziz University, PO Box 80203 Jeddah, Saudi Arabia

## Abstract

Two independent but virtually identical mol­ecules comprise the asymmetric unit in the title compound, C_18_H_15_NOS. With reference to the quinolin-3-yl group, the 3-(thio­phen-2-yl)prop-2-en-1-one residue is almost perpendicular, with all but the carbonyl O atom lying to one side of the plane. This conformation is reflected by the C—C—C—C torsion angles of −102.2 (3) and 81.1 (3)° in the two independent mol­ecules. The dihedral angle formed between the 13 non-H atoms directly associated with the quinolin-3-yl group and the thio­phen-2-yl ring is 87.70 (11)° [83.85 (10)° for the second independent mol­ecule]. The presence of C—H⋯O, C—H⋯N and π–π inter­actions [centroid–centroid distance = 3.5590 (12) Å] lead to supra­molecular chains along the *c*-axis direction. These are connected along the *a*-axis direction by C—H⋯π inter­actions. The resultant supra­molecular layers stack along the *b* axis.

## Related literature

For background details and biological applications of quinolines, see: Kalluraya & Sreenivasa (1998 ▶); Xiang *et al.* (2006 ▶). For the biological activity of chalcones, see: Dimmock *et al.* (1999 ▶); Siddiqui *et al.* (2008 ▶).
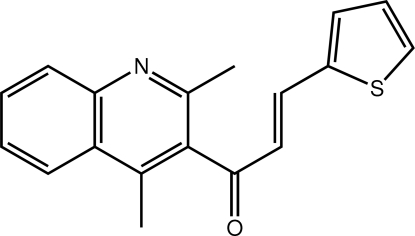


## Experimental

### 

#### Crystal data

C_18_H_15_NOS
*M* *_r_* = 293.37
Monoclinic, 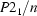

*a* = 10.4935 (4) Å
*b* = 23.8464 (8) Å
*c* = 11.5464 (4) Å
β = 93.756 (3)°
*V* = 2883.07 (18) Å^3^
*Z* = 8
Mo *K*α radiation
μ = 0.22 mm^−1^
*T* = 100 K
0.25 × 0.20 × 0.15 mm

#### Data collection

Agilent SuperNova Dual diffractometer with an Atlas detector
Absorption correction: multi-scan (*CrysAlis PRO*; Agilent, 2010 ▶) *T*_min_ = 0.947, *T*_max_ = 0.967
14916 measured reflections
6427 independent reflections
4075 reflections with *I* > 2σ(*I*)
*R*_int_ = 0.040

#### Refinement

*R*[*F* ^2^ > 2σ(*F* ^2^)] = 0.055
*wR*(*F* ^2^) = 0.160
*S* = 1.03
6427 reflections
409 parameters
182 restraints
H-atom parameters constrained
Δρ_max_ = 0.56 e Å^−3^
Δρ_min_ = −0.46 e Å^−3^

### 

Data collection: *CrysAlis PRO* (Agilent, 2010 ▶); cell refinement: *CrysAlis PRO*; data reduction: *CrysAlis PRO*; program(s) used to solve structure: *SHELXS97* (Sheldrick, 2008 ▶); program(s) used to refine structure: *SHELXL97* (Sheldrick, 2008 ▶); molecular graphics: *ORTEP-3* (Farrugia, 1997 ▶), *DIAMOND* (Brandenburg, 2006 ▶) and *Qmol* (Gans & Shalloway, 2001 ▶); software used to prepare material for publication: *publCIF* (Westrip, 2010 ▶).

## Supplementary Material

Crystal structure: contains datablock(s) global, I. DOI: 10.1107/S1600536811031485/hg5075sup1.cif

Structure factors: contains datablock(s) I. DOI: 10.1107/S1600536811031485/hg5075Isup2.hkl

Supplementary material file. DOI: 10.1107/S1600536811031485/hg5075Isup3.cml

Additional supplementary materials:  crystallographic information; 3D view; checkCIF report

## Figures and Tables

**Table 1:** Hydrogen-bond geometry (Å, °) *Cg*1 and *Cg*2 are the centroids of the C31–C36 and C13–C18 rings, respectively.

*D*—H⋯*A*	*D*—H	H⋯*A*	*D*⋯*A*	*D*—H⋯*A*
C5—H5⋯N2^i^	0.95	2.51	3.391 (3)	154
C6—H6⋯O2	0.95	2.55	3.382 (3)	146
C23—H23⋯N1^ii^	0.95	2.50	3.382 (3)	154
C24—H24⋯O1	0.95	2.48	3.330 (3)	149
C12—H12c⋯*Cg*1^iii^	0.98	2.67	142	4 (1)
C26—H26c⋯*Cg*2^iv^	0.98	2.67	143	4 (1)

Symmetry codes: (i) 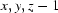; (ii) 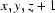; (iii) 
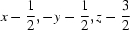
; (iv) 
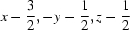
.

**Fig. 1.**
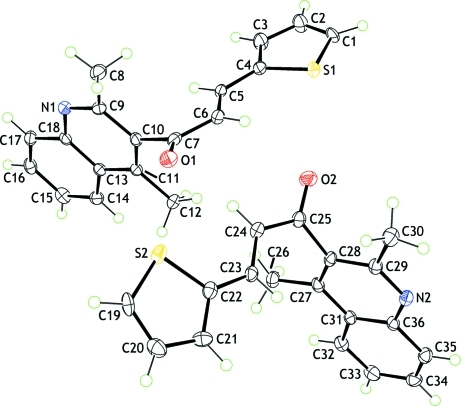
The molecular structures of the two independent molecules comprising the asymmetric unit of (I) showing the atom-labelling scheme and displacement ellipsoids at the 50% probability level.

**Fig. 2.**
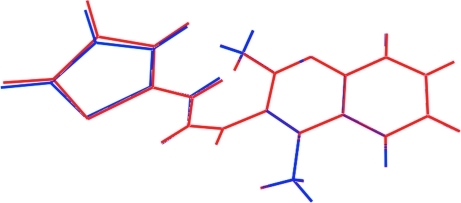
Overlay diagram of the two independent molecules comprising the asymmetric unit of (I). The first independent molecule (with the S1 atom) is shown in red.

**Fig. 3.**
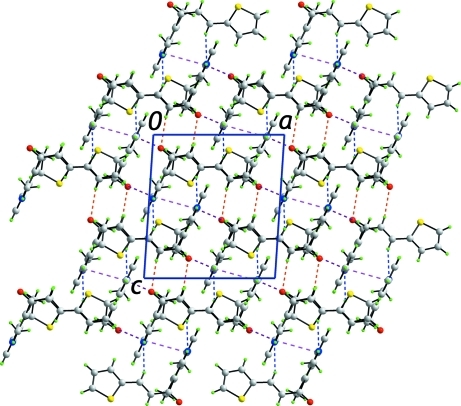
Assembly of molecules in the ac plane in (I) mediated by C—H···O (orange dashed lines), C—H···N (blue), C—H···π (purple) and π–π (pink) interactions.

**Fig. 4.**
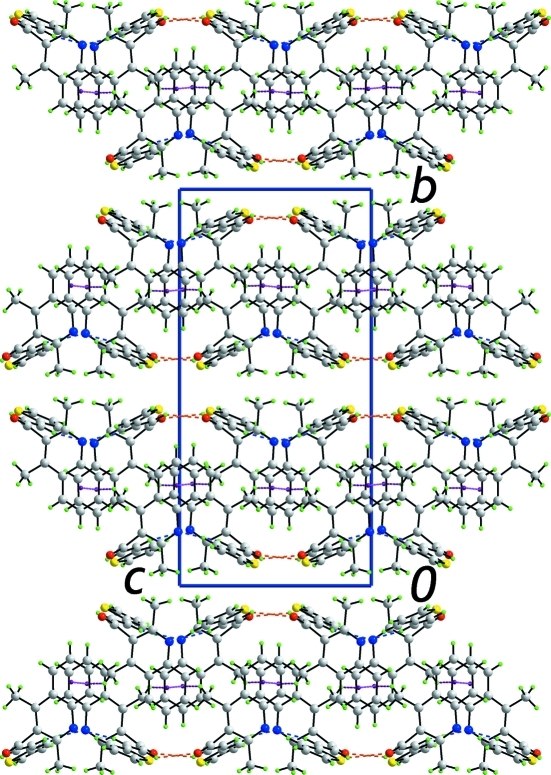
A view in projection down the a axis of the crystal packing in (I) highlighting the stacking of layers along the b axis.

## supplementary crystallographic information

### Comment

Chalcone derivatives have attracted wide attention owing to their occurrence in natural products and biologically active compounds (Dimmock *et al.*, 1999; Siddiqui *et al.*, 2008). Quinoline chalcone analogues have also gained notice due to their bioactivities such as anti-plasmodial, anti-microbial, anti-malarial and anti-cancer activities (Kalluraya & Sreenivasa, 1998; Xiang *et al.*, 2006). It was in this context that the title compound, (I), was investigated.

Two independent molecules comprise the crystallographic asymmetric unit of (I), Fig. 1, and as may be seen from Fig. 2, the molecular conformations are almost identical with a minor variation in the relative orientations of the terminal thiophenyl rings. The 3-(thiophen-2-yl)prop-2-en-1-one residue is almost normal to the least-squares plane through the quinolin-3-yl group and three bound C substituents (r.m.s. deviations = 0.028 and 0.035 Å, respectively), *i.e*. 13 non-C atoms. This conformation is reflected in the C6—C7—C10—C9 and C24—C25—C28—C27 torsion angles of -102.2 (3) and 81.1 (3) °, respectively. With reference to the aforementioned quinolin-3-yl plane, the carbonyl-O lies to one side and the remaining atoms of the 3-(thiophen-2-yl)prop-2-en-1-one residue to the other. The dihedral angles formed between the quinolin-3-yl and thiophen-2-yl rings are 87.70 (11) and 83.85 (10) °, respectively. The conformation about the ethene bond is *E* [C5═C6 = 1.345 (3) Å, and C23═C24 = 1.341 (3) Å].

In the crystal packing, supramolecular layers are formed in the *ac* plane owing to a combination of C—H···O, C—H···N, C—H···π and π–π interactions, Table 1 and Fig. 3. The two independent molecules comprising the asymmetric unit are linked *via* the C—H···O interactions, to form an eight-membered {···O═C_2_H}_2_ synthon. These are linked into a supramolecular chain along the *c* axis by C—H···N and π–π interactions. The C—H···π contacts extend in the *a* direction. Layers stack along the *b* axis as illustrated in Fig. 4.

### Experimental

A mixture of 3-acetyl-2,4-dimethylquinoline (0.01 *M*), 2-thiophenecarboxaldehyde (0.01 *M*) and a catalytic amount of KOH in distilled ethanol was stirred for 12 h at room temperature. The resulting mixture was neutralized with dilute acetic acid. The solid that formed was filtered, dried and purified by column chromatography using 1:3 mixture of ethyl acetate and hexane. Recrystallization was by slow evaporation of its acetone solution which yielded colourless needles. *M*.pt. 418–420 K. Yield: 82%.

### Refinement

Carbon-bound H-atoms were placed in calculated positions [C—H 0.95 to 0.98 Å, *U*_iso_(H) 1.2 to 1.5*U*_eq_(C)] and were included in the refinement in the riding model approximation. Each thiophen-2-yl ring was found to be disordered so that there were two co-planar but oppositely orientated orientations for each. Pairs of 1,2- and 1,3-related distances involving the unprimed and primed atoms were restrained to within 0.01 Å of each other. Each ring was restrained to lie on a plane within 0.01 Å. The anisotropic displacement factors of the primed atoms were set to those of the unprimed ones, and were restrained to be nearly isotropic. From the refinement, the major component of the S1- and S2-containing rings were 0.765 (2) and 0.814 (2), respectively.

### Crystal data

**Table:**

C_18_H_15_NOS	*F*(000) = 1232
*M**_r_* = 293.37	*D*_x_ = 1.352 Mg m^−^^3^
Monoclinic, *P*2_1_/*n*	Mo *K*α radiation, λ = 0.71073 Å
Hall symbol: -P 2yn	Cell parameters from 4109 reflections
*a* = 10.4935 (4) Å	θ = 2.5–29.2°
*b* = 23.8464 (8) Å	µ = 0.22 mm^−^^1^
*c* = 11.5464 (4) Å	*T* = 100 K
β = 93.756 (3)°	Block, colourless
*V* = 2883.07 (18) Å^3^	0.25 × 0.20 × 0.15 mm
*Z* = 8	

### Data collection

**Table:**

Agilent SuperNova Dual diffractometer with an Atlas detector	6427 independent reflections
Radiation source: SuperNova (Mo) X-ray Source	4075 reflections with *I* > 2σ(*I*)
Mirror	*R*_int_ = 0.040
Detector resolution: 10.4041 pixels mm^-1^	θ_max_ = 27.5°, θ_min_ = 2.5°
ω scans	*h* = −13→13
Absorption correction: multi-scan (*CrysAlis PRO*; Agilent, 2010)	*k* = −30→22
*T*_min_ = 0.947, *T*_max_ = 0.967	*l* = −14→11
14916 measured reflections	

### Refinement

**Table:**

Refinement on *F*^2^	Primary atom site location: structure-invariant direct methods
Least-squares matrix: full	Secondary atom site location: difference Fourier map
*R*[*F*^2^ > 2σ(*F*^2^)] = 0.055	Hydrogen site location: inferred from neighbouring sites
*wR*(*F*^2^) = 0.160	H-atom parameters constrained
*S* = 1.03	*w* = 1/[σ^2^(*F*_o_^2^) + (0.0713*P*)^2^ + 0.299*P*] where *P* = (*F*_o_^2^ + 2*F*_c_^2^)/3
6427 reflections	(Δ/σ)_max_ < 0.001
409 parameters	Δρ_max_ = 0.56 e Å^−^^3^
182 restraints	Δρ_min_ = −0.46 e Å^−^^3^

### Special details

**Table:**

Geometry. All s.u.'s (except the s.u. in the dihedral angle between two l.s. planes) are estimated using the full covariance matrix. The cell s.u.'s are taken into account individually in the estimation of s.u.'s in distances, angles and torsion angles; correlations between s.u.'s in cell parameters are only used when they are defined by crystal symmetry. An approximate (isotropic) treatment of cell s.u.'s is used for estimating s.u.'s involving l.s. planes.
Refinement. Refinement of *F*^2^ against ALL reflections. The weighted *R*-factor *wR* and goodness of fit *S* are based on *F*^2^, conventional *R*-factors *R* are based on *F*, with *F* set to zero for negative *F*^2^. The threshold expression of *F*^2^ > 2σ(*F*^2^) is used only for calculating *R*-factors(gt) *etc*. and is not relevant to the choice of reflections for refinement. *R*-factors based on *F*^2^ are statistically about twice as large as those based on *F*, and *R*- factors based on ALL data will be even larger.

### Fractional atomic coordinates and isotropic or equivalent isotropic displacement parameters (Å^2^)

**Table:**

	*x*	*y*	*z*	*U*_iso_*/*U*_eq_	Occ. (<1)
S1	0.30670 (9)	0.05958 (3)	0.34043 (8)	0.0224 (3)	0.765 (2)
S2	1.08225 (8)	0.05541 (3)	0.60970 (7)	0.0228 (3)	0.814 (2)
S1'	0.2891 (5)	0.10382 (16)	0.1135 (4)	0.0224 (3)	0.235 (2)
S2'	1.1144 (6)	0.10712 (19)	0.8273 (5)	0.0228 (3)	0.186 (2)
O1	0.83016 (16)	0.07720 (7)	0.36269 (14)	0.0240 (4)	
O2	0.56274 (16)	0.07794 (7)	0.59472 (14)	0.0259 (4)	
N1	0.87096 (18)	0.13639 (8)	0.00826 (16)	0.0182 (4)	
N2	0.52776 (18)	0.14083 (8)	0.94581 (16)	0.0181 (4)	
C1	0.1610 (3)	0.06381 (10)	0.2659 (3)	0.0246 (9)	0.765 (2)
H1	0.0835	0.0521	0.2965	0.029*	0.765 (2)
C2	0.1682 (4)	0.08583 (10)	0.1576 (3)	0.0284 (9)	0.765 (2)
H2	0.0971	0.0912	0.1035	0.034*	0.765 (2)
C3	0.2925 (5)	0.09927 (16)	0.1371 (4)	0.0253 (14)	0.765 (2)
H3	0.3144	0.1157	0.0661	0.030*	0.765 (2)
C1'	0.1608 (11)	0.0803 (2)	0.1824 (9)	0.0246 (9)	0.235 (2)
H1'	0.0755	0.0803	0.1492	0.029*	0.235 (2)
C2'	0.1964 (10)	0.0612 (2)	0.2923 (9)	0.0284 (9)	0.235 (2)
H2'	0.1379	0.0468	0.3442	0.034*	0.235 (2)
C3'	0.3256 (10)	0.0652 (2)	0.3189 (7)	0.0253 (14)	0.235 (2)
H3'	0.3678	0.0542	0.3906	0.030*	0.235 (2)
C4	0.3847 (2)	0.08728 (9)	0.22700 (19)	0.0194 (5)	
C5	0.5200 (2)	0.09698 (9)	0.22462 (19)	0.0176 (5)	
H5	0.5482	0.1144	0.1569	0.021*	
C6	0.6106 (2)	0.08403 (10)	0.3082 (2)	0.0202 (5)	
H6	0.5863	0.0667	0.3776	0.024*	
C7	0.7457 (2)	0.09599 (9)	0.29473 (19)	0.0179 (5)	
C8	0.8360 (2)	0.04453 (10)	0.0864 (2)	0.0245 (6)	
H8A	0.8668	0.0341	0.0111	0.037*	
H8B	0.7505	0.0289	0.0934	0.037*	
H8C	0.8944	0.0296	0.1487	0.037*	
C9	0.8305 (2)	0.10735 (10)	0.09594 (19)	0.0176 (5)	
C10	0.7826 (2)	0.13304 (9)	0.19578 (19)	0.0173 (5)	
C11	0.7760 (2)	0.19024 (9)	0.20381 (19)	0.0168 (5)	
C12	0.7250 (2)	0.21978 (10)	0.3063 (2)	0.0202 (5)	
H12A	0.6995	0.1920	0.3628	0.030*	
H12B	0.6507	0.2425	0.2802	0.030*	
H12C	0.7915	0.2441	0.3426	0.030*	
C13	0.8209 (2)	0.22287 (9)	0.11100 (19)	0.0164 (5)	
C14	0.8211 (2)	0.28242 (10)	0.1100 (2)	0.0204 (5)	
H14	0.7912	0.3024	0.1740	0.024*	
C15	0.8636 (2)	0.31132 (10)	0.0179 (2)	0.0221 (5)	
H15	0.8635	0.3512	0.0185	0.027*	
C16	0.9074 (2)	0.28255 (10)	−0.0774 (2)	0.0213 (5)	
H16	0.9358	0.3030	−0.1415	0.026*	
C17	0.9096 (2)	0.22560 (10)	−0.0792 (2)	0.0198 (5)	
H17	0.9405	0.2067	−0.1441	0.024*	
C18	0.8664 (2)	0.19410 (9)	0.01481 (19)	0.0162 (5)	
C19	1.2324 (3)	0.06405 (10)	0.6756 (3)	0.0227 (8)	0.814 (2)
H19	1.3088	0.0523	0.6429	0.027*	0.814 (2)
C20	1.2283 (3)	0.08988 (9)	0.7808 (3)	0.0262 (8)	0.814 (2)
H20	1.3016	0.0983	0.8304	0.031*	0.814 (2)
C21	1.1044 (5)	0.10228 (15)	0.8064 (4)	0.0231 (12)	0.814 (2)
H21	1.0851	0.1205	0.8763	0.028*	0.814 (2)
C19'	1.2382 (12)	0.0828 (3)	0.7536 (10)	0.0227 (8)	0.186 (2)
H19B	1.3253	0.0851	0.7812	0.027*	0.186 (2)
C20'	1.1962 (11)	0.0594 (3)	0.6493 (9)	0.0262 (8)	0.186 (2)
H20B	1.2514	0.0437	0.5959	0.031*	0.186 (2)
C21'	1.0652 (12)	0.0613 (2)	0.6307 (7)	0.0231 (12)	0.186 (2)
H21B	1.0189	0.0473	0.5633	0.028*	0.186 (2)
C22	1.0104 (2)	0.08626 (9)	0.72294 (19)	0.0186 (5)	
C23	0.8752 (2)	0.09631 (9)	0.7276 (2)	0.0172 (5)	
H23	0.8484	0.1127	0.7970	0.021*	
C24	0.7833 (2)	0.08503 (9)	0.6447 (2)	0.0185 (5)	
H24	0.8061	0.0684	0.5742	0.022*	
C25	0.6489 (2)	0.09748 (9)	0.6595 (2)	0.0184 (5)	
C26	0.6714 (2)	0.22170 (10)	0.6441 (2)	0.0211 (5)	
H26A	0.6954	0.1934	0.5880	0.032*	
H26B	0.7465	0.2441	0.6692	0.032*	
H26C	0.6054	0.2463	0.6078	0.032*	
C27	0.6204 (2)	0.19306 (10)	0.74765 (19)	0.0173 (5)	
C28	0.6142 (2)	0.13577 (9)	0.75760 (19)	0.0160 (5)	
C29	0.5674 (2)	0.11100 (10)	0.85886 (19)	0.0176 (5)	
C30	0.5630 (2)	0.04845 (9)	0.8706 (2)	0.0239 (6)	
H30A	0.5345	0.0386	0.9471	0.036*	
H30B	0.6484	0.0329	0.8623	0.036*	
H30C	0.5033	0.0329	0.8101	0.036*	
C31	0.5763 (2)	0.22622 (9)	0.84016 (19)	0.0161 (5)	
C32	0.5758 (2)	0.28574 (10)	0.8390 (2)	0.0201 (5)	
H32	0.6059	0.3052	0.7744	0.024*	
C33	0.5327 (2)	0.31534 (10)	0.9295 (2)	0.0233 (6)	
H33	0.5322	0.3552	0.9271	0.028*	
C34	0.4888 (2)	0.28715 (10)	1.0269 (2)	0.0227 (6)	
H34	0.4601	0.3081	1.0901	0.027*	
C35	0.4877 (2)	0.22986 (10)	1.0305 (2)	0.0203 (5)	
H35	0.4573	0.2112	1.0960	0.024*	
C36	0.5314 (2)	0.19812 (9)	0.93734 (19)	0.0163 (5)	

### Atomic displacement parameters (Å^2^)

**Table:**

	*U*^11^	*U*^22^	*U*^33^	*U*^12^	*U*^13^	*U*^23^
S1	0.0241 (5)	0.0243 (5)	0.0195 (5)	−0.0060 (4)	0.0075 (3)	−0.0004 (3)
S2	0.0227 (5)	0.0262 (5)	0.0201 (4)	0.0063 (3)	0.0063 (3)	−0.0009 (3)
S1'	0.0241 (5)	0.0243 (5)	0.0195 (5)	−0.0060 (4)	0.0075 (3)	−0.0004 (3)
S2'	0.0227 (5)	0.0262 (5)	0.0201 (4)	0.0063 (3)	0.0063 (3)	−0.0009 (3)
O1	0.0256 (10)	0.0285 (10)	0.0174 (9)	0.0003 (8)	−0.0019 (8)	0.0032 (7)
O2	0.0213 (9)	0.0341 (10)	0.0220 (9)	−0.0001 (8)	−0.0015 (8)	−0.0057 (8)
N1	0.0164 (10)	0.0207 (10)	0.0176 (10)	−0.0011 (8)	0.0014 (8)	−0.0021 (8)
N2	0.0173 (10)	0.0211 (10)	0.0160 (10)	0.0017 (8)	0.0027 (8)	0.0009 (8)
C1	0.0109 (15)	0.0289 (18)	0.034 (2)	−0.0063 (14)	0.0056 (16)	−0.0078 (16)
C2	0.0205 (18)	0.034 (2)	0.031 (2)	−0.0014 (15)	0.0037 (16)	0.0022 (16)
C3	0.023 (2)	0.035 (2)	0.018 (3)	−0.0026 (16)	−0.0002 (19)	0.0067 (18)
C1'	0.0109 (15)	0.0289 (18)	0.034 (2)	−0.0063 (14)	0.0056 (16)	−0.0078 (16)
C2'	0.0205 (18)	0.034 (2)	0.031 (2)	−0.0014 (15)	0.0037 (16)	0.0022 (16)
C3'	0.023 (2)	0.035 (2)	0.018 (3)	−0.0026 (16)	−0.0002 (19)	0.0067 (18)
C4	0.0207 (13)	0.0170 (12)	0.0211 (13)	−0.0017 (10)	0.0062 (10)	−0.0041 (10)
C5	0.0215 (13)	0.0170 (11)	0.0151 (11)	−0.0038 (10)	0.0071 (10)	−0.0024 (9)
C6	0.0235 (13)	0.0211 (12)	0.0168 (12)	−0.0040 (10)	0.0064 (10)	−0.0020 (10)
C7	0.0234 (13)	0.0170 (12)	0.0136 (11)	−0.0003 (10)	0.0034 (10)	−0.0026 (9)
C8	0.0302 (14)	0.0206 (13)	0.0234 (13)	−0.0020 (11)	0.0065 (11)	−0.0035 (10)
C9	0.0172 (12)	0.0215 (12)	0.0141 (11)	−0.0014 (10)	−0.0005 (10)	−0.0014 (9)
C10	0.0129 (12)	0.0242 (12)	0.0147 (11)	−0.0028 (10)	0.0008 (9)	0.0007 (9)
C11	0.0112 (11)	0.0236 (12)	0.0157 (11)	−0.0009 (10)	0.0003 (9)	−0.0030 (10)
C12	0.0203 (13)	0.0225 (12)	0.0183 (12)	0.0009 (10)	0.0053 (10)	−0.0037 (10)
C13	0.0114 (11)	0.0202 (12)	0.0173 (12)	0.0001 (9)	−0.0009 (9)	−0.0010 (9)
C14	0.0161 (12)	0.0215 (13)	0.0234 (13)	0.0018 (10)	0.0002 (10)	−0.0043 (10)
C15	0.0189 (13)	0.0192 (12)	0.0279 (14)	0.0017 (10)	−0.0009 (11)	0.0047 (10)
C16	0.0169 (13)	0.0269 (13)	0.0202 (13)	−0.0002 (10)	0.0016 (10)	0.0074 (10)
C17	0.0167 (12)	0.0260 (13)	0.0168 (12)	−0.0005 (10)	0.0030 (10)	0.0001 (10)
C18	0.0108 (11)	0.0218 (12)	0.0159 (12)	−0.0025 (10)	−0.0003 (9)	0.0000 (9)
C19	0.0129 (15)	0.0265 (17)	0.0296 (19)	0.0053 (13)	0.0086 (14)	0.0095 (14)
C20	0.0179 (16)	0.0256 (16)	0.035 (2)	0.0010 (13)	−0.0009 (15)	−0.0017 (14)
C21	0.024 (2)	0.0246 (19)	0.020 (2)	0.0005 (14)	−0.0058 (17)	−0.0020 (16)
C19'	0.0129 (15)	0.0265 (17)	0.0296 (19)	0.0053 (13)	0.0086 (14)	0.0095 (14)
C20'	0.0179 (16)	0.0256 (16)	0.035 (2)	0.0010 (13)	−0.0009 (15)	−0.0017 (14)
C21'	0.024 (2)	0.0246 (19)	0.020 (2)	0.0005 (14)	−0.0058 (17)	−0.0020 (16)
C22	0.0215 (13)	0.0156 (11)	0.0192 (12)	0.0012 (10)	0.0052 (10)	0.0023 (9)
C23	0.0202 (13)	0.0155 (11)	0.0164 (12)	−0.0013 (10)	0.0059 (10)	0.0014 (9)
C24	0.0197 (13)	0.0208 (12)	0.0154 (12)	0.0019 (10)	0.0038 (10)	0.0001 (10)
C25	0.0200 (13)	0.0197 (12)	0.0155 (12)	0.0002 (10)	0.0025 (10)	0.0031 (9)
C26	0.0209 (13)	0.0250 (13)	0.0179 (12)	−0.0004 (10)	0.0043 (10)	0.0030 (10)
C27	0.0119 (12)	0.0227 (12)	0.0171 (12)	−0.0015 (10)	−0.0001 (9)	0.0032 (10)
C28	0.0114 (11)	0.0228 (12)	0.0141 (11)	0.0039 (9)	0.0016 (9)	−0.0001 (9)
C29	0.0143 (12)	0.0218 (12)	0.0168 (12)	0.0016 (10)	0.0012 (9)	0.0023 (10)
C30	0.0313 (14)	0.0176 (12)	0.0232 (13)	0.0005 (11)	0.0064 (11)	0.0031 (10)
C31	0.0108 (11)	0.0196 (12)	0.0180 (12)	0.0007 (9)	0.0017 (9)	−0.0009 (9)
C32	0.0178 (13)	0.0209 (12)	0.0216 (13)	−0.0017 (10)	0.0013 (10)	0.0032 (10)
C33	0.0179 (13)	0.0189 (12)	0.0330 (15)	0.0002 (10)	0.0011 (11)	−0.0040 (11)
C34	0.0179 (13)	0.0265 (13)	0.0237 (13)	0.0011 (10)	0.0016 (10)	−0.0090 (11)
C35	0.0167 (12)	0.0250 (13)	0.0195 (13)	0.0024 (10)	0.0027 (10)	−0.0010 (10)
C36	0.0109 (11)	0.0218 (12)	0.0163 (12)	0.0010 (9)	0.0011 (9)	0.0003 (10)

### Geometric parameters (Å, °)

**Table:**

S1—C1	1.708 (3)	C14—H14	0.9500
S1—C4	1.721 (2)	C15—C16	1.399 (3)
S2—C22	1.717 (2)	C15—H15	0.9500
S2—C19	1.717 (3)	C16—C17	1.358 (3)
S1'—C4	1.646 (5)	C16—H16	0.9500
S1'—C1'	1.704 (8)	C17—C18	1.418 (3)
S2'—C22	1.649 (6)	C17—H17	0.9500
S2'—C19'	1.700 (8)	C19—C20	1.365 (4)
O1—C7	1.229 (3)	C19—H19	0.9500
O2—C25	1.227 (3)	C20—C21	1.384 (5)
N1—C9	1.319 (3)	C20—H20	0.9500
N1—C18	1.379 (3)	C21—C22	1.387 (4)
N2—C29	1.320 (3)	C21—H21	0.9500
N2—C36	1.370 (3)	C19'—C20'	1.374 (8)
C1—C2	1.362 (4)	C19'—H19B	0.9500
C1—H1	0.9500	C20'—C21'	1.379 (8)
C2—C3	1.379 (5)	C20'—H20B	0.9500
C2—H2	0.9500	C21'—C22	1.378 (7)
C3—C4	1.401 (5)	C21'—H21B	0.9500
C3—H3	0.9500	C22—C23	1.443 (3)
C1'—C2'	1.377 (8)	C23—C24	1.341 (3)
C1'—H1'	0.9500	C23—H23	0.9500
C2'—C3'	1.373 (8)	C24—C25	1.461 (3)
C2'—H2'	0.9500	C24—H24	0.9500
C3'—C4	1.369 (7)	C25—C28	1.517 (3)
C3'—H3'	0.9500	C26—C27	1.505 (3)
C4—C5	1.440 (3)	C26—H26A	0.9800
C5—C6	1.345 (3)	C26—H26B	0.9800
C5—H5	0.9500	C26—H26C	0.9800
C6—C7	1.465 (3)	C27—C28	1.373 (3)
C6—H6	0.9500	C27—C31	1.430 (3)
C7—C10	1.515 (3)	C28—C29	1.426 (3)
C8—C9	1.503 (3)	C29—C30	1.499 (3)
C8—H8A	0.9800	C30—H30A	0.9800
C8—H8B	0.9800	C30—H30B	0.9800
C8—H8C	0.9800	C30—H30C	0.9800
C9—C10	1.426 (3)	C31—C36	1.414 (3)
C10—C11	1.369 (3)	C31—C32	1.420 (3)
C11—C13	1.429 (3)	C32—C33	1.362 (3)
C11—C12	1.505 (3)	C32—H32	0.9500
C12—H12A	0.9800	C33—C34	1.413 (3)
C12—H12B	0.9800	C33—H33	0.9500
C12—H12C	0.9800	C34—C35	1.367 (3)
C13—C18	1.415 (3)	C34—H34	0.9500
C13—C14	1.420 (3)	C35—C36	1.416 (3)
C14—C15	1.367 (3)	C35—H35	0.9500
			
C1—S1—C4	92.77 (15)	N1—C18—C13	122.8 (2)
C22—S2—C19	92.72 (14)	N1—C18—C17	118.2 (2)
C4—S1'—C1'	90.5 (5)	C13—C18—C17	119.0 (2)
C22—S2'—C19'	91.3 (5)	C20—C19—S2	111.5 (2)
C9—N1—C18	117.9 (2)	C20—C19—H19	124.2
C29—N2—C36	118.1 (2)	S2—C19—H19	124.2
C2—C1—S1	112.5 (3)	C19—C20—C21	111.8 (3)
C2—C1—H1	123.7	C19—C20—H20	124.1
S1—C1—H1	123.7	C21—C20—H20	124.1
C1—C2—C3	110.9 (3)	C22—C21—C20	115.3 (4)
C1—C2—H2	124.5	C22—C21—H21	122.3
C3—C2—H2	124.5	C20—C21—H21	122.3
C2—C3—C4	116.1 (4)	C20'—C19'—S2'	111.5 (8)
C2—C3—H3	121.9	C20'—C19'—H19B	124.3
C4—C3—H3	121.9	S2'—C19'—H19B	124.3
C2'—C1'—S1'	111.3 (8)	C19'—C20'—C21'	112.5 (7)
C2'—C1'—H1'	124.3	C19'—C20'—H20B	123.8
S1'—C1'—H1'	124.3	C21'—C20'—H20B	123.8
C3'—C2'—C1'	112.8 (7)	C20'—C21'—C22	110.8 (6)
C3'—C2'—H2'	123.6	C20'—C21'—H21B	124.6
C1'—C2'—H2'	123.6	C22—C21'—H21B	124.6
C2'—C3'—C4	110.2 (6)	C21—C22—C21'	110.0 (6)
C2'—C3'—H3'	124.9	C21—C22—C23	125.8 (3)
C4—C3'—H3'	124.9	C21'—C22—C23	124.2 (6)
C3—C4—C3'	109.1 (6)	C21—C22—S2'	3.9 (4)
C3—C4—C5	125.9 (3)	C21'—C22—S2'	113.9 (5)
C3'—C4—C5	125.0 (5)	C23—C22—S2'	121.9 (3)
C3—C4—S1'	6.1 (4)	C21—C22—S2	108.6 (3)
C3'—C4—S1'	115.2 (5)	C21'—C22—S2	1.4 (5)
C5—C4—S1'	119.8 (3)	C23—C22—S2	125.55 (17)
C3—C4—S1	107.6 (3)	S2'—C22—S2	112.5 (3)
C3'—C4—S1	1.5 (5)	C24—C23—C22	127.1 (2)
C5—C4—S1	126.48 (17)	C24—C23—H23	116.5
S1'—C4—S1	113.7 (2)	C22—C23—H23	116.5
C6—C5—C4	126.9 (2)	C23—C24—C25	122.0 (2)
C6—C5—H5	116.6	C23—C24—H24	119.0
C4—C5—H5	116.6	C25—C24—H24	119.0
C5—C6—C7	121.5 (2)	O2—C25—C24	122.0 (2)
C5—C6—H6	119.3	O2—C25—C28	118.7 (2)
C7—C6—H6	119.3	C24—C25—C28	119.36 (19)
O1—C7—C6	121.6 (2)	C27—C26—H26A	109.5
O1—C7—C10	119.0 (2)	C27—C26—H26B	109.5
C6—C7—C10	119.41 (19)	H26A—C26—H26B	109.5
C9—C8—H8A	109.5	C27—C26—H26C	109.5
C9—C8—H8B	109.5	H26A—C26—H26C	109.5
H8A—C8—H8B	109.5	H26B—C26—H26C	109.5
C9—C8—H8C	109.5	C28—C27—C31	117.9 (2)
H8A—C8—H8C	109.5	C28—C27—C26	122.6 (2)
H8B—C8—H8C	109.5	C31—C27—C26	119.4 (2)
N1—C9—C10	122.9 (2)	C27—C28—C29	120.1 (2)
N1—C9—C8	116.8 (2)	C27—C28—C25	121.4 (2)
C10—C9—C8	120.3 (2)	C29—C28—C25	118.4 (2)
C11—C10—C9	120.3 (2)	N2—C29—C28	122.9 (2)
C11—C10—C7	120.9 (2)	N2—C29—C30	117.0 (2)
C9—C10—C7	118.7 (2)	C28—C29—C30	120.1 (2)
C10—C11—C13	118.1 (2)	C29—C30—H30A	109.5
C10—C11—C12	122.8 (2)	C29—C30—H30B	109.5
C13—C11—C12	119.1 (2)	H30A—C30—H30B	109.5
C11—C12—H12A	109.5	C29—C30—H30C	109.5
C11—C12—H12B	109.5	H30A—C30—H30C	109.5
H12A—C12—H12B	109.5	H30B—C30—H30C	109.5
C11—C12—H12C	109.5	C36—C31—C32	118.7 (2)
H12A—C12—H12C	109.5	C36—C31—C27	118.2 (2)
H12B—C12—H12C	109.5	C32—C31—C27	123.2 (2)
C18—C13—C14	118.5 (2)	C33—C32—C31	120.8 (2)
C18—C13—C11	118.0 (2)	C33—C32—H32	119.6
C14—C13—C11	123.5 (2)	C31—C32—H32	119.6
C15—C14—C13	120.7 (2)	C32—C33—C34	120.4 (2)
C15—C14—H14	119.6	C32—C33—H33	119.8
C13—C14—H14	119.6	C34—C33—H33	119.8
C14—C15—C16	120.4 (2)	C35—C34—C33	120.3 (2)
C14—C15—H15	119.8	C35—C34—H34	119.9
C16—C15—H15	119.8	C33—C34—H34	119.9
C17—C16—C15	120.6 (2)	C34—C35—C36	120.5 (2)
C17—C16—H16	119.7	C34—C35—H35	119.8
C15—C16—H16	119.7	C36—C35—H35	119.8
C16—C17—C18	120.8 (2)	N2—C36—C31	122.8 (2)
C16—C17—H17	119.6	N2—C36—C35	117.8 (2)
C18—C17—H17	119.6	C31—C36—C35	119.4 (2)
			
C4—S1—C1—C2	−0.36 (11)	C22—S2—C19—C20	−0.20 (10)
S1—C1—C2—C3	−0.33 (19)	S2—C19—C20—C21	−0.04 (19)
C1—C2—C3—C4	1.1 (3)	C19—C20—C21—C22	0.3 (3)
C4—S1'—C1'—C2'	1.0 (4)	C22—S2'—C19'—C20'	0.5 (4)
S1'—C1'—C2'—C3'	−0.7 (6)	S2'—C19'—C20'—C21'	−0.2 (6)
C1'—C2'—C3'—C4	−0.2 (6)	C19'—C20'—C21'—C22	−0.3 (6)
C2—C3—C4—C3'	−1.4 (4)	C20—C21—C22—C21'	−0.5 (4)
C2—C3—C4—C5	179.2 (2)	C20—C21—C22—C23	−177.6 (2)
C2—C3—C4—S1	−1.3 (3)	C20—C21—C22—S2	−0.5 (3)
C2'—C3'—C4—C3	0.9 (5)	C20'—C21'—C22—C21	0.4 (5)
C2'—C3'—C4—C5	−179.6 (3)	C20'—C21'—C22—C23	177.6 (3)
C2'—C3'—C4—S1'	1.0 (5)	C20'—C21'—C22—S2'	0.6 (5)
C2'—C3'—C4—S1	−1(8)	C20'—C21'—C22—S2	−1(8)
C1'—S1'—C4—C3	−1(2)	C19'—S2'—C22—C21	2(4)
C1'—S1'—C4—C3'	−1.2 (4)	C19'—S2'—C22—C21'	−0.6 (4)
C1'—S1'—C4—C5	179.4 (2)	C19'—S2'—C22—C23	−177.7 (2)
C1'—S1'—C4—S1	−1.1 (3)	C19'—S2'—C22—S2	−0.6 (3)
C1—S1—C4—C3	0.9 (2)	C19—S2—C22—C21	0.38 (19)
C1—S1—C4—C5	−179.6 (2)	C19—S2—C22—C23	177.5 (2)
C1—S1—C4—S1'	0.96 (18)	C19—S2—C22—S2'	0.55 (19)
C3—C4—C5—C6	−177.5 (3)	C21—C22—C23—C24	174.7 (3)
C3'—C4—C5—C6	3.1 (4)	C21'—C22—C23—C24	−2.0 (4)
S1'—C4—C5—C6	−177.5 (2)	S2'—C22—C23—C24	174.8 (2)
S1—C4—C5—C6	3.1 (4)	S2—C22—C23—C24	−2.0 (3)
C4—C5—C6—C7	179.7 (2)	C22—C23—C24—C25	−179.7 (2)
C5—C6—C7—O1	−169.4 (2)	C23—C24—C25—O2	−165.9 (2)
C5—C6—C7—C10	11.5 (3)	C23—C24—C25—C28	14.3 (3)
C18—N1—C9—C10	0.1 (3)	C31—C27—C28—C29	−1.2 (3)
C18—N1—C9—C8	179.80 (19)	C26—C27—C28—C29	178.7 (2)
N1—C9—C10—C11	0.5 (3)	C31—C27—C28—C25	175.06 (19)
C8—C9—C10—C11	−179.1 (2)	C26—C27—C28—C25	−5.0 (3)
N1—C9—C10—C7	−176.5 (2)	O2—C25—C28—C27	−98.7 (3)
C8—C9—C10—C7	3.9 (3)	C24—C25—C28—C27	81.1 (3)
O1—C7—C10—C11	−98.3 (3)	O2—C25—C28—C29	77.7 (3)
C6—C7—C10—C11	80.8 (3)	C24—C25—C28—C29	−102.5 (3)
O1—C7—C10—C9	78.7 (3)	C36—N2—C29—C28	0.4 (3)
C6—C7—C10—C9	−102.2 (3)	C36—N2—C29—C30	179.72 (19)
C9—C10—C11—C13	−1.4 (3)	C27—C28—C29—N2	0.4 (3)
C7—C10—C11—C13	175.53 (19)	C25—C28—C29—N2	−176.0 (2)
C9—C10—C11—C12	179.3 (2)	C27—C28—C29—C30	−178.8 (2)
C7—C10—C11—C12	−3.8 (3)	C25—C28—C29—C30	4.7 (3)
C10—C11—C13—C18	1.7 (3)	C28—C27—C31—C36	1.2 (3)
C12—C11—C13—C18	−178.99 (19)	C26—C27—C31—C36	−178.75 (19)
C10—C11—C13—C14	−178.9 (2)	C28—C27—C31—C32	−178.9 (2)
C12—C11—C13—C14	0.4 (3)	C26—C27—C31—C32	1.2 (3)
C18—C13—C14—C15	0.3 (3)	C36—C31—C32—C33	−0.1 (3)
C11—C13—C14—C15	−179.1 (2)	C27—C31—C32—C33	180.0 (2)
C13—C14—C15—C16	0.3 (3)	C31—C32—C33—C34	0.6 (3)
C14—C15—C16—C17	−0.8 (3)	C32—C33—C34—C35	−0.9 (3)
C15—C16—C17—C18	0.7 (3)	C33—C34—C35—C36	0.6 (3)
C9—N1—C18—C13	0.2 (3)	C29—N2—C36—C31	−0.4 (3)
C9—N1—C18—C17	−179.94 (19)	C29—N2—C36—C35	179.47 (19)
C14—C13—C18—N1	179.5 (2)	C32—C31—C36—N2	179.7 (2)
C11—C13—C18—N1	−1.1 (3)	C27—C31—C36—N2	−0.4 (3)
C14—C13—C18—C17	−0.4 (3)	C32—C31—C36—C35	−0.2 (3)
C11—C13—C18—C17	179.03 (19)	C27—C31—C36—C35	179.71 (19)
C16—C17—C18—N1	−180.0 (2)	C34—C35—C36—N2	−179.9 (2)
C16—C17—C18—C13	−0.1 (3)	C34—C35—C36—C31	0.0 (3)

### Hydrogen-bond geometry (Å, °)

**Table:**

Cg1 and Cg2 are the centroids of the C31–C36 and C13–C18 rings, respectively.

**Table:**

*D*—H···*A*	*D*—H	H···*A*	*D*···*A*	*D*—H···*A*
C5—H5···N2^i^	0.95	2.51	3.391 (3)	154
C6—H6···O2	0.95	2.55	3.382 (3)	146
C23—H23···N1^ii^	0.95	2.50	3.382 (3)	154
C24—H24···O1	0.95	2.48	3.330 (3)	149
C12—H12c···Cg1^iii^	0.98	2.67	142	3.499 (2)
C26—H26c···Cg2^iv^	0.98	2.67	143	3.503 (2)

Symmetry codes: (i) *x*, *y*, *z*−1; (ii) *x*, *y*, *z*+1; (iii) *x*−1/2, −*y*−1/2, *z*−3/2; (iv) *x*−3/2, −*y*−1/2, *z*−1/2.

## References

[bb1] Agilent (2010). *CrysAlis PRO* Agilent Technologies, Yarnton, England.

[bb2] Brandenburg, K. (2006). *DIAMOND* Crystal Impact GbR, Bonn, Germany.

[bb3] Dimmock, J. R., Elias, D. W., Beazely, M. A. & Kandepu, N. M. (1999). *Curr. Med. Chem.* **6**, 1125–1149. 10519918

[bb4] Farrugia, L. J. (1997). *J. Appl. Cryst.* **30**, 565.

[bb5] Gans, J. & Shalloway, D. (2001). *J. Mol. Graph. Model.* **19**, 557–559. 10.1016/s1093-3263(01)00090-0 11552684

[bb6] Kalluraya, B. & Sreenivasa, S. (1998). *Farmaco*, **53**, 399–404. 10.1016/s0014-827x(98)00037-8 9764472

[bb7] Sheldrick, G. M. (2008). *Acta Cryst.* A**64**, 112–122. 10.1107/S0108767307043930 18156677

[bb8] Siddiqui, Z. B., Asad, M. & Praveen, S. (2008). *Med. Chem. Res.* **17**, 318–325.

[bb9] Westrip, S. P. (2010). *J. Appl. Cryst.* **43**, 920–925.

[bb10] Xiang, W., Tiekink, E. R. T., Iouri, K., Nikolai, K. & Mei, L. G. (2006). *Eur. J. Pharm. Sci.* **27**, 175–187.

